# Efficient Electrolytic Refining of Crude Solder Assisted by Additives in a Fluosilicic Acid System

**DOI:** 10.3390/ma18174122

**Published:** 2025-09-02

**Authors:** Yuantao Yang, Zhaoyi Wang, Xuanbing Wang, Wanli Xu, Haibin Yuan, Qingdong Liu, Ruidong Xu, Linjing Yang

**Affiliations:** 1 State Key Laboratory of Complex Nonferrous Metal Resources Clean Utilization, Kunming University of Science and Technology, Kunming 650093, China; 20222102065@stu.kust.edu.cn (Y.Y.); wangzhaoyi@stu.kust.edu.cn (Z.W.); wangxuanbing@stu.kust.edu.cn (X.W.); 2 Faculty of Metallurgical and Energy Engineering, Kunming University of Science and Technology, Kunming 650093, China; 3 Yunnan Tin Company Group Limited, Kunming 650200, China; 20222102056@stu.kust.edu.cn (W.X.); 20222102050@stu.kust.edu.cn (H.Y.); liuqingdong@stu.kust.edu.cn (Q.L.)

**Keywords:** solder electrolytic refining, additives, electrochemical behavior, element flow direction

## Abstract

Current electrolytic refining processes for crude solder commonly employ fluosilicic acid (H_2_SiF_6_) as the electrolyte with bone glue and β-naphthol additives yet suffer from poor electrolyte stability, coarse cathode crystallization, low current efficiency, and high energy consumption, adversely affecting product quality and economic viability. In order to solve these limitations, electrochemical techniques, XRD, SEM, and ICP-OES were used to study the effects of gelatin and sodium lignosulfonate on the deposition overpotential and cathode morphology, as well as the effects of process parameters on current efficiency and energy consumption. A novel approach was developed using an H_2_SiF_6_ system enhanced by gelatin and sodium lignosulfonate for crude solder refining. After optimization, 120 h electrolysis achieved a current efficiency >97.8%, smooth/dense cathode surface, average cell voltage of 0.24 V, and energy consumption of 98.15 kWh/t. Efficient deposition of 81.2% Sn and 75.2% Pb on the cathode was realized, while >93.3% of Sb, Bi, Ag, Cu, and As were enriched in anode slime to facilitate valuable metal recovery, and >90.6% of In/Al concentrated in the electrolyte enabled effective Sn-Pb impurity separation. This study provides theoretical and technical foundations for advancing sustainable and economical electrolytic refining of crude solder.

## 1. Introduction

Tin, characterized by its high electrical conductivity, excellent solderability, corrosion resistance, stable performance, and high specific capacity and energy density, is an indispensable key metal in modern industry [1,2,3,4]. It is widely used in various fields such as electronics, information technology, electrical appliances, chemicals, metallurgy, building materials, machinery, and food packaging [5,6,7]. The refining of tin is carried out through several methods, including pyrometallurgical refining [8], vacuum distillation [9], and electrolytic refining [10]. Pyrometallurgical refining involves a series of operations, such as centrifugation and condensation, to remove arsenic and iron, the addition of sulfur to remove copper, and the addition of aluminum to remove arsenic and antimony, with continuous crystallization to remove lead and bismuth. Although the majority of tin is refined through pyrometallurgical processes, these require multiple coordinated operations to achieve impurity removal. Vacuum distillation of tin can overcome some of the drawbacks associated with atmospheric pressure pyrometallurgical refining, but it demands high-quality equipment and regular inspection and maintenance of the furnace. Electrolytic refining, on the other hand, has the advantage of removing most impurities from crude solder in a single operation and producing high-purity solder, making it suitable for large-scale production [11].

In solder electrolytic refining, the differences in the ease of dissolution or deposition of various elements during the electrolysis process are utilized. Tin and lead are dissolved at the anode and then deposited at the cathode, while other impurities remain in the anode slime or electrolyte. Current solder electrolytic refining processes typically use fluosilicic acid (H_2_SiF_6_) as the electrolyte and add bone glue and β-naphthol as additives to optimize the refining process. However, this process suffers from poor electrolyte stability, coarse crystallization of cathode solder, low current efficiency, and high energy consumption, which severely impact product quality and process economic viability. To address these issues, the optimization of process parameters through the use of electrolytes and additives is primarily focused on improving the electrolysis effect, with suitable additives yielding smooth and dense deposits and enhancing current efficiency.

Recently, Xiang et al. [12] employed methanesulfonic acid as the electrolyte and β-naphthol ethoxylate (Lugalvan BNO12) as an auxiliary additive for the electrolytic refining of crude lead, achieving lead with a purity greater than 99.99% and a smooth, dense appearance. The laboratory-scale energy demand was 87 kWh/t Pb with a cathode current efficiency of 99.6%. Chang et al. [13,14,15] used anion membrane electrolysis technology, adding calcium lignosulfonate to methanesulfonic acid electrolyte to efficiently produce metallic bismuth with a purity exceeding 99.96%, achieving a current efficiency of 98.80% and an energy consumption of 712.53 kWh/t Bi. Zhang et al. [16] refined solder using methanesulfonic acid electrolyte, with an average cell voltage of 0.30 V and a current efficiency of 99.31%, yielding high-purity solder with Sn and Pb content greater than 99.99% and good product appearance. Current research is mainly focused on the use of environmentally friendly electrolytes, such as methanesulfonic acid [17,18,19]. However, methanesulfonic acid is mostly at the experimental stage, and its electrolyte cost is much higher than that of H_2_SiF_6_ [12]. Therefore, enterprises using H_2_SiF_6_ as the electrolyte for solder electrolytic refining cannot replace H_2_SiF_6_ with methanesulfonic acid in the short term.

Based on this, this study investigates the combined use of gelatin and sodium lignosulfonate in a H_2_SiF_6_ system. Using electrochemical techniques, the effects of these additives on deposition overpotential are studied. This study systematically investigates the effects of Sn^2+^/Pb^2+^ concentration, H_2_SiF_6_ concentration, current density, and electrolysis temperature on current efficiency, cell voltage, and energy consumption in the electrolytic refining of crude solder. By optimizing process parameters, the distribution and flow of elements during electrolysis are elucidated, effectively processing crude solder produced from tin smelting and the reduction melting of lead–tin coexisting ores. This study provides theoretical support for further improving the current efficiency and product quality of solder electrolytic refining and optimizing the refining process.

## 2. Materials and Methods

### 2.1. Experimental Principles

**Figure 1:**
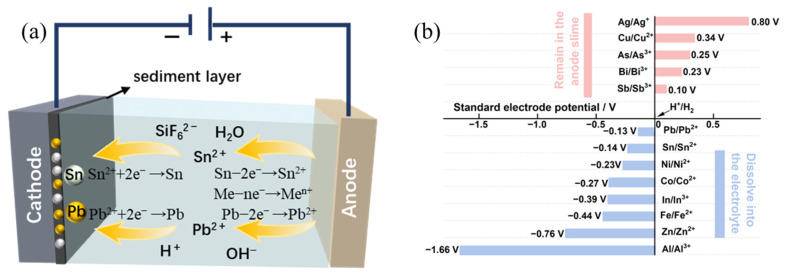
Electrolytic refining: (**a**) schematic diagram, (**b**) element standard electrode potential.

The principle of electrolytic refining of solder in fluorosilicic acid system is shown in Figure 1. According to the element standard electrode potential Figure 1b, the main reactions involved are as follows:

Anode reaction

Sn − 2e^−^ → Sn^2+^ (1)

Pb − 2e^−^→ Pb^2+^ (2)

Me^−^ ne^−^ → Me^n+^; Me: In, Fe, Ni, Zn… (3)

Cathodic reaction

Sn^2+^ + 2e^−^→Sn (4)

Pb^2+^ + 2e^−^ → Pb (5)

The electrolytic refining of solder in a H_2_SiF_6_ system involves three stages: anode dissolution, ion migration, and cathode electrodeposition. During the anode dissolution stage, Sn and Pb dissolve into the electrolyte via reactions (1) and (2). Impurity elements with standard electrode potentials more negative than those of Sn and Pb (such as In, Fe, Zn, etc.) dissolve into the electrolyte as their respective metal ions through reaction (3). In contrast, impurity elements with standard electrode potentials more positive than Sn (such as Sb, Bi, Ag, etc.) essentially remain unreacted and are retained in the anode slime. During the ion migration stage, driven by the electric field, the principal metal ions migrate towards the surface of the cathode plate. In the cathode deposition stage, Sn^2+^ and Pb^2+^ are reduced and deposited on the cathode plate through reactions (4) and (5). Ions, such as In, Fe, and Zn, which have much higher reduction potentials than Sn^2+^, remain in the electrolyte. Additionally, although the standard electrode potential for the reduction in hydrogen ions to form H_2_ is more positive than that of Sn and Pb, the significant overvoltage associated with hydrogen evolution at the cathode shifts its reduction potential to a more negative value. This overvoltage effect results in a negative potential for hydrogen evolution that is more negative than the standard electrode potentials of Sn and Pb, thereby enabling the preferential reduction and deposition of Sn^2+^ and Pb^2+^ over hydrogen ions at the cathode.

The calculation formula of the electrochemical equivalent of tin–lead alloy is as follows:

(6) $$q_{Sn-Pb}=\frac{1}{\frac{x_{Sn}\%}{q_{Sn}}+\frac{x_{Pb}\%}{q_{Pb}}}$$

In the formula, $q_{Sn-Pb}$ is the electrochemical equivalent of tin–lead alloy, g/(A·h); $q_{Sn}$ is the electrochemical equivalent of tin, 2.214 g/(A·h); $q_{Pb}$ is the electrochemical equivalent of lead, 3.865 g/(A·h); and $x_{Sn}$% and $x_{Pb}$% are the proportion of tin and lead content in solder.

The calculation formula of cathode current efficiency is as follows:

(7) $$\eta =\frac{∆m}{q_{Sn-Pb}\times I\times t}\times 100\%$$

In the formula, η is the current efficiency, %; ∆m is the quality of solder deposited on the cathode, g; I is the current through, A; and t is the electrolysis time, h.

The power consumption is calculated according to the following formula:

(8) $$W=\frac{1000\times U}{\eta \times q_{Sn-Pb}}\times 100\%$$

In the formula, W is the power consumption, kWh/t, and u is the cell voltage, V.

### 2.2. Materials

All reagents, including H_2_SiF_6_, stannous oxide (SnO), lead oxide (PbO), gelatin, and alumina polishing powder, were purchased from Aladdin (Shanghai, China). Bone glue and β-naphthol were sourced from Yunnan Tin Company Group Limited (Kunming, China). Deionized water was prepared in the laboratory. The chemical composition of the crude solder is presented in Table 1.

### 2.3. Experimental Equipment

**Figure 2:**
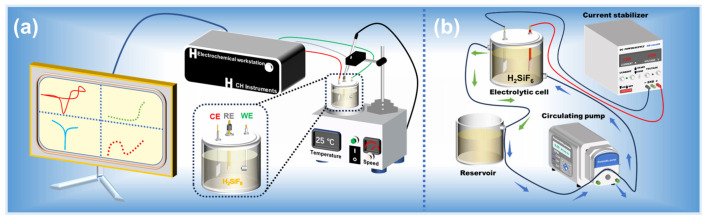
Experimental equipment and test process 3D schematic diagram: (**a**) electrochemical experiments, (**b**) electrolytic refining experiment.

In this study, the electrochemical testing was conducted using an electrochemical workstation (CHI 760E, Shanghai Chenhua Instruments Co., Ltd. Shanghai, China). The setup for the electrodeposition experiment is illustrated in Figure 2a. A 3 mm diameter “L”-shaped glassy carbon electrode was employed as the working electrode, while a platinum sheet (1 × 1 cm^2^) and a saturated calomel electrode were used as the counter electrode and reference electrode, respectively. To prevent cross-contamination between the electrodes and the electrolyte, a salt bridge filled with saturated KCl solution was utilized. Prior to each test, the working electrode was pre-treated, polished to a mirror finish with ultra-fine Al_2_O_3_ slurry, and then ultrasonically cleaned with distilled water to ensure the cleanliness of the electrode surface.

For the electrolytic refining section, as shown in Figure 2b, a rectifier was used to supply the current. The electrolyte circulation system, equipped with a temperature control unit and a peristaltic pump, facilitated the electrolytic refining experiments, which were designed to optimize the solder electrolytic refining process.

## 3. Results and Discussion

### 3.1. Electrochemical Behavior of Metal Deposition

In the process of metal electrodeposition, the nucleation overpotential (NOP), defined as NOP = φ_CO_ − φ_dep_ (where φ_CO_ is a crossover potential and φ_dep_ is the deposition potential), is commonly used to quantify the polarization degree of the cathode metal [13,20,21,22]. A larger NOP value indicates a higher degree of polarization, resulting in a smoother cathode surface morphology and smaller grain sizes.

**Figure 3:**
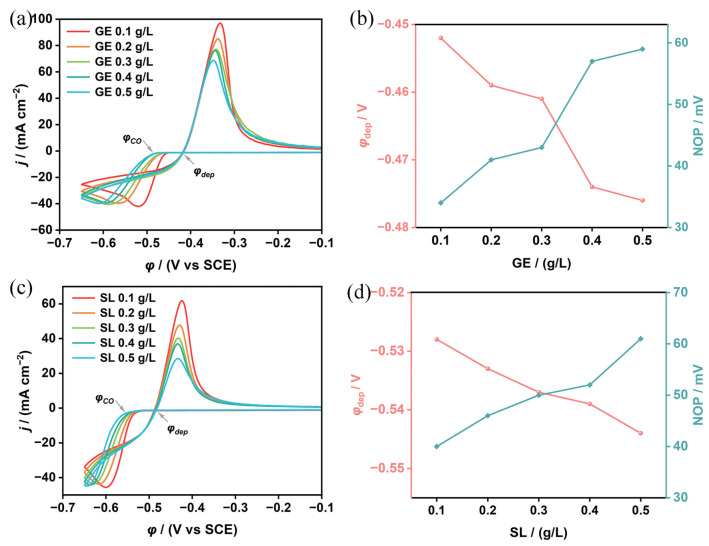
The principle of electrodeposition in gelatin-assisted fluorosilicic acid system: (**a**) cyclic voltammogram, (**b**) the change in deposition overpotential and nucleation overpotential; the principle of electrodeposition in sodium lignosulfonate-assisted fluorosilicic acid system, (**c**) cyclic voltammograms, (**d**) change diagrams of deposition overpotential and nucleation overpotential.

To investigate the effects of gelatin (GE) and sodium lignosulfonate (SL) on the deposition behavior of tin and lead on the glassy carbon electrode in a H_2_SiF_6_ system, cyclic voltammogram curves were obtained for various concentrations of gelatin and sodium lignosulfonate, as shown in Figure 3. The cathodic portion of the cyclic voltammograms (Figure 3a,c) reveals a nucleation loop, where the current during the reverse scan is higher than that during the forward scan at the same overpotential. This indicates that the initial deposition of Sn^2+^ involves nucleation and growth processes [23,24,25]. The relevant data, including deposition overpotential, reduction peak potential, and equilibrium potential, were extracted from Figure 3a,c and are presented in Tables S1 and S2. As shown in Figure 3a,b and Table S1, there is an increase in concentration of gelatin from 0.1 g/L to 0.5 g/L, which results in a negative shift in φ_dep_ and a decrease in the reduction peak current density (j_pc_) from 41.9 mA/cm^2^ to 39.8 mA/cm^2^, suggesting that gelatin inhibits the electrodeposition of Sn and Pb. This inhibition is likely due to the adsorption of gelatin on the electrode surface, which alters the surface charge distribution and increases the polarization of the electrode. This increased polarization makes the deposition of Sn and Pb ions more difficult, requiring a higher overpotential, thus causing a negative shift in the deposition overpotential and a decrease in the reduction peak current density.

As shown in Figure 3c,d and Table S2, as the increase of sodium lignosulfonate from 0.1 g/L to 0.5 g/L results in a negative shift in φ_dep_ from −0.528 V to −0.544 V and in φ_pc_ from −0.600 V to −0.645 V, with j_pc_ decreasing from 45.6 mA/cm^2^ to 44.8 mA/cm^2^, suggesting that the sodium lignosulfonate also inhibits the electrodeposition of Sn and Pb. Sodium lignosulfonate molecules, enriched with sulfonic and hydroxyl functional groups, adsorb strongly onto the electrode surface, forming a stable interfacial layer. This adsorption layer modulates surface charge distribution, blocks active sites, and consequently inhibits metal ion deposition. The resultant charge transfer barrier significantly increases deposition overpotential. Collectively, these findings demonstrate that both gelatin and sodium lignosulfonate effectively suppress Sn and Pb deposition kinetics.

### 3.2. Cathode Surface Morphology

**Figure 4:**
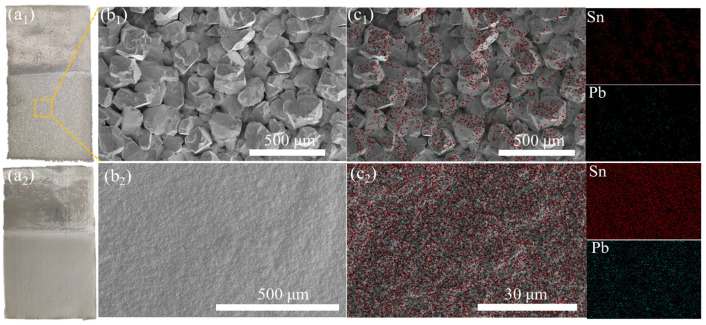
Cathodic deposition morphology: (**a_1_**,**b_1_**,**c_1_**) no additives, (**a_2_**,**b_2_**,**c_2_**) combination of gelatin and sodium lignosulfonate.

In the absence of additives, the morphology and energy-dispersive X-ray spectroscopy (EDS) of the cathode surface are shown in Figure 4(a_1_,b_1_,c_1_). Without additives, the cathode surface appears relatively rough, characterized by small granular structures. As depicted in the scanning electron microscopy (SEM) image in Figure 4(b_1_), the cathode exhibits a non-dense, blocky growth with significant inter-particle gaps. The EDS distribution map in Figure 4(c_1_) indicates a uniform distribution of Sn and Pb on the surface.

In contrast, when gelatin and sodium lignosulfonate are used in conjunction with H_2_SiF_6_, the cathode plate formation is shown in Figure 4(a_2_,b_2_,c_2_). Compared to the scenario without additives, the cathode plate exhibits fewer protrusions and a smoother, denser, and more lustrous surface. As illustrated in Figure 4(c_2_), Sn and Pb are uniformly distributed across the cathode plate, meeting the morphological requirements for cathode plates in solder electrolytic refining processes.

### 3.3. Evaluation of Cathode Current Efficiency and Energy Consumption in Solder Electrolytic Refining

**Figure 5:**
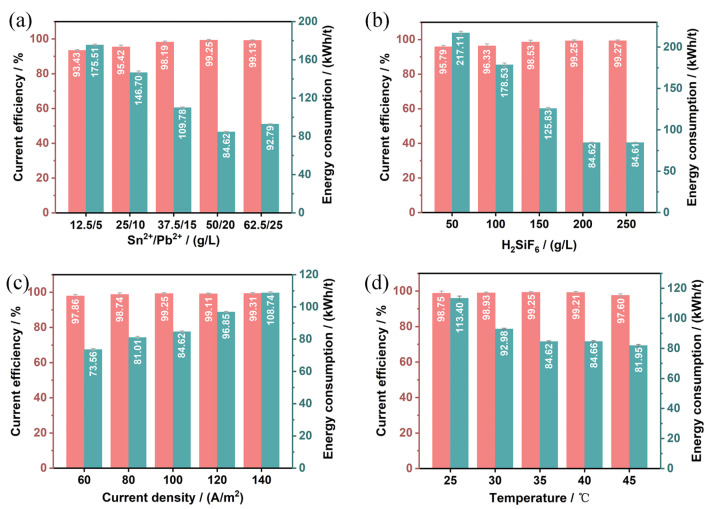
Cathodic current efficiency and energy consumption of crude solder under different conditions: (**a**) Sn^2+^/Pb^2+^ concentration; (**b**) H_2_SiF_6_ concentration; (**c**) current density; (**d**) temperature.

The cathode current efficiency and energy consumption in the electrolytic refining of crude solder were evaluated to further optimize the solder electrolytic refining process. The effects of current density, Sn^2+^/Pb^2+^ concentration, H_2_SiF_6_ concentration, and temperature on current efficiency and energy consumption are shown in Figure 5, while their effects on cell voltage are presented in Figure S1.

The effects of Sn^2+^ and Pb^2+^ concentrations on current efficiency, energy consumption, and cell voltage during the electrolytic refining are illustrated in Figure 5a and Figure S1a. As the concentrations of Sn^2+^ and Pb^2+^ increase, the current efficiency also rises. Specifically, when the concentrations of Sn^2+^ and Pb^2+^ are increased from 12.5 g/L and 5 g/L to 50 g/L and 20 g/L, respectively, the current efficiency increases from 93.43% to 99.25%, the cell voltage decreases from 0.31 V to 0.21 V, and the energy consumption drops from 132.70 kWh/t to 84.62 kWh/t. This improvement is primarily due to the reduced difference in ion concentration between the electrode surface and the bulk solution, which lowers the resistance to the reduction reactions of Sn and Pb and increases the resistance to hydrogen evolution, thereby enhancing current efficiency [19]. However, further increasing the concentrations of Sn^2+^ and Pb^2+^ to 62.5 g/L and 25 g/L results in a current efficiency of 99.13%, with no further improvement. This is likely because the interactions between ions in the electrolyte are enhanced at higher ion concentrations, which may hinder ion migration. Additionally, excessively high ion concentrations can increase the viscosity of the electrolyte, thereby limiting further increases in current efficiency. Moreover, when the concentrations of Sn^2+^ and Pb^2+^ are excessively high, the dissolution rate of Sn^2+^ and Pb^2+^ at the anode surface may exceed their diffusion rate in the electrolyte, leading to the formation of a concentration polarization layer rich in Sn^2+^ and Pb^2+^ on the anode surface. This layer can impede the dissolution of metal ions at the anode, exacerbate anode passivation, and increase the activation energy required for the electrode reaction. As a result, higher voltages and energy consumption are needed to sustain the anode reaction, leading to increased cell voltage and energy consumption. Therefore, considering these factors, concentrations of 50 g/L for Sn^2+^ and 20 g/L for Pb^2+^ are deemed optimal.

The impact of H_2_SiF_6_ concentration on current efficiency, cell voltage, and energy consumption during the electrolytic refining of crude solder is depicted in Figure 5b and Figure S1b. As the concentration of H_2_SiF_6_ increases from 50 g/L to 250 g/L, the current efficiency rises from 95.79% to 99.27%, the cell voltage decreases from 0.52 V to 0.21 V, and the energy consumption drops from 217.11 kWh/t to 84.60 kWh/t. The increase in acid concentration leads to a higher hydrogen ion concentration in the electrolyte, which enhances the electrolyte’s conductivity and facilitates easier current transmission, thereby reducing the solution’s resistance and minimizing energy losses due to resistance. However, when the concentration of H_2_SiF_6_ is further increased from 200 g/L to 250 g/L, there is little change in current efficiency, cell voltage, and energy consumption. Excessively high acid concentrations may lower the overpotential for hydrogen evolution, increasing the likelihood of hydrogen evolution as a side reaction at the cathode [26]. Therefore, an H_2_SiF_6_ concentration of 200 g/L is selected as the optimal value.

The influence of current density on current efficiency, cell voltage, and energy consumption during the electrolytic refining of crude solder is shown in Figure 5c and Figure S1c. As the current density increases from 60 A/m^2^ to 100 A/m^2^, the current efficiency rises from 97.86% to 99.25%. This increase is due to the higher current density, which accelerates the reduction rate of Sn and Pb ions at the cathode surface, enhances the deposition rate, and reduces interference from impurity ions and side reactions, thereby improving current efficiency. However, further increasing the current density beyond 100 A/m^2^ does not result in further improvements in current efficiency. As shown in Figure S1c, higher current densities lead to increased cell voltage and energy consumption. This is because the increased current density results in greater ohmic voltage drops in the electrolyte, electrodes, and contact points, collectively contributing to higher total voltage and energy consumption [27]. Therefore, a current density of 100 A/m^2^ is chosen as the optimal value.

The impact of temperature on current efficiency, cell voltage, and energy consumption during the electrolytic refining of crude solder is shown in Figure 5d and Figure S1d. As the temperature increases from 25 °C to 40 °C, the current efficiency rises from 98.75% to 99.21%. This improvement is attributed to the increased conductivity of the electrolyte at higher temperatures, which reduces energy losses due to resistance. Additionally, the increased thermal motion of ions in the electrolyte at higher temperatures reduces the diffusion and migration resistance of ions, facilitating faster oxidation-reduction reactions at the electrode surface and thereby enhancing current efficiency [28,29]. However, further increasing the temperature to 45 °C increases the likelihood of impurities in the electrolyte participating in reactions, which can consume some electrical energy and lower current efficiency [30]. Moreover, higher temperatures accelerate the evaporation of the electrolyte, altering the ion concentration and acidity and worsening the working environment due to acid volatilization [31]. As shown in Figure S1d, the cell voltage decreases from 0.28 V to 0.2 V as the temperature increases from 25 °C to 45 °C, but the rate of decrease in cell voltage slows down when the temperature exceeds 35 °C. Considering the overall electrolytic performance and electrolyte loss, an electrolytic temperature of 35 °C is selected.

### 3.4. Expansion Experiment of Electrolytic Refining of Solder

#### 3.4.1. Cathodic Deposition Morphology

To verify the scalability of the optimized process, an expanded validation experiment was conducted with the following parameters: Sn^2+^ and Pb^2+^ concentrations were set at 50 g/L and 20 g/L, respectively; H_2_SiF_6_ concentration was maintained at 200 g/L; current density was controlled at 100 A/m^2^; and the electrolysis temperature was kept at 35 °C. The cathode morphology after 24 h and 48 h of electrolysis is shown in Figure S2. After 24 h of electrolysis, the cathode surface was smooth and dense. However, after extending the electrolysis to 48 h, dendritic growth was observed at the edges of the cathode plate. This phenomenon was primarily attributed to the consumption of additives in the electrolyte over time. To address this issue, the process was modified to replenish the additives every 24 h.

**Figure 6:**
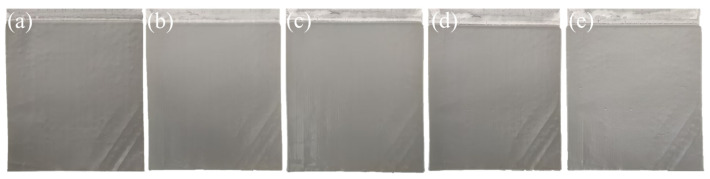
The changes of cathode morphology with the extension of electrolysis time: (**a**) 24 h, (**b**) 48 h, (**c**) 72 h, (**d**) 96 h, (**e**) 120 h.

Following the implementation of this adjustment, the cathode surface morphology was monitored over extended electrolysis periods, as depicted in Figure 6. The results indicated that the cathode surface remained smooth and dense throughout the electrolysis process. Even after 96 h of electrolysis, only a few small particle protrusions appeared on the cathode surface, and these did not significantly increase in size, even after 120 h. This stable morphology meets the requirements for cathode appearance in solder electrolytic refining processes.

#### 3.4.2. Proof of Concept Operation of Crude Solder Electrorefining

**Figure 7:**
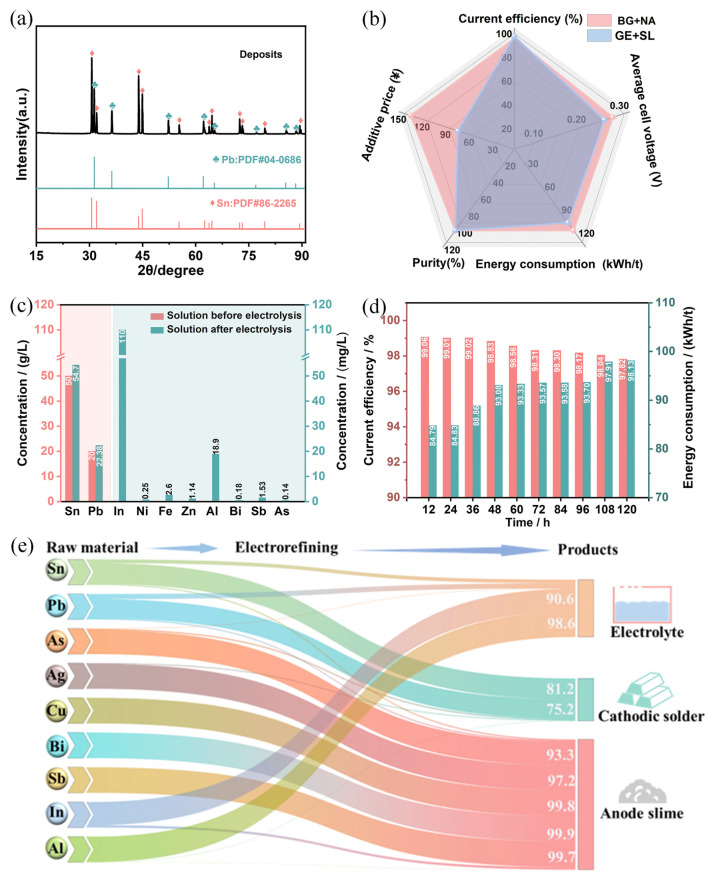
Laboratory-scale operation and economic evaluation of electrorefining of crude solder in gelatin and sodium lignosulphonate-assisted H_2_SiF_6_ system: (**a**) cathode plate XRD, (**b**) comparison between gelatin and sodium lignosulphonate-assisted H_2_SiF_6_ system and bone glue and β-naphthol-assisted H_2_SiF_6_ system, (**c**) evolution of electrolyte composition, (**d**) variation of cathode current efficiency and energy consumption with electrolysis time, (**e**) element distribution behavior after electrolysis.

To verify the technical and economic feasibility of using a combination of gelatin and sodium lignosulfonate in a H_2_SiF_6_ system for the electrolytic refining of crude solder, we conducted long-term, scaled-up experimental studies, with the results presented in Figure 7.

After the completion of electrolysis, XRD analysis of the cathode plate, as shown in Figure 7a, revealed diffraction peaks that matched the standard PDF cards (86-2265 for Sn and 04-0686 for Pb), indicating that the deposits on the cathode plate were pure Sn and Pb. Additionally, ICP-OES analysis of the cathode plate, detailed in Table S3, showed that the total content of Sn and Pb was 99.98%. The impurity content included 0.0012% Bi, 0.003% Sb, 0.0019% As, and 0.00069% Ag, while other impurities such as In, Cu, Ni, Zn, and Fe were present in trace amounts, all below 0.0005%. XPS analysis of the cathode plate is shown in Figure S3. It can be seen in the figure that the valence states of tin and lead are zero and positive tetravalent, respectively, indicating that the main forms of tin and lead are tin–lead alloys and oxides. The reason for the presence of oxides is the inevitable oxidation of surfaces exposed to air [32,33].

As illustrated in the radar chart in Figure 7b, the combination of gelatin and sodium lignosulfonate (GE + SL) achieved a current efficiency of 97.8%, surpassing the 96.1% obtained with bone glue and β-naphthol (BG + NA). The cell voltage and energy consumption for GE + SL were 0.24 V and 98.14 kWh/t, respectively, both lower than the 0.26 V and 108.21 kWh/t for BG + NA. Regarding additive costs, bone glue ranged from 20 to 50 CNY/kg, β-naphthol from 50 to 150 CNY/kg, sodium lignosulfonate from 5 to 15 CNY/kg, and gelatin from 30 to 100 CNY/kg. The BG + NA combination was slightly more expensive than GE + SL and required additional steam for dissolution, incurring extra energy consumption in practical production. In terms of electrolyte selection, methanesulfonic acid and fluorosilicic acid are compared as shown in Table S4. Although MSA has more advantages in green environmental protection, its cost is much higher than that of H_2_SiF_6_. The fluorosilicic acid electrolyte currently used by enterprises cannot be replaced in a short time.

The compositional changes of the electrolyte before and after electrolysis are depicted in Figure 7c. Post-electrolysis, the concentration of Sn increased from 50 g/L to 54.7 g/L, and that of Pb rose from 20 g/L to 22.38 g/L. The increase in the concentrations of the principal metals Sn and Pb in the electrolyte can be attributed to the higher dissolution rate at the anode compared to the deposition rate at the cathode, resulting in incomplete recovery of the dissolved metal ions at the cathode. Additionally, evaporation losses during the electrolysis process contributed to the elevated levels of Sn and Pb. Metals with standard electrode potentials more negative than Sn and Pb, such as In, Ni, Fe, Zn, and Al, accumulated in the electrolyte. Conversely, metals with standard electrode potentials more positive than Sn and Pb, like Bi, Sb, and As, which were present in larger quantities in the raw material, had minute fractions migrating into the electrolyte. This facilitated the separation of impurities from Sn and Pb, which is beneficial for subsequent centralized treatment. As shown in Figure S4, the compositional changes in the electrolyte after multiple cycles of use indicate that the concentrations of these impurities did not significantly increase, owing to the electrolyte loss during electrolysis and the replenishment of fresh electrolytes. Therefore, from the perspectives of resource utilization and cost-effectiveness, this electrolyte demonstrates potential for cyclic use.

As shown in Figure 7d and Figure S5, current efficiency decreased with prolonged electrolysis time. At 12 h of electrolysis, the current efficiency was 99.06%, with a cell voltage and energy consumption of 0.21 V and 84.83 kWh/t, respectively. After extending the electrolysis to 48 h, the current efficiency dropped to 98.8%, and the cell voltage and energy consumption rose to 0.23 V and 93.10 kWh/t. Comparing these results with those without additive replenishment (Table S5) revealed that periodic replenishment of additives every 24 h improved the electrolysis performance. After 120 h of electrolysis, the current efficiency decreased to 97.8%. The average cell voltage increased from 0.21 V at 12 h to 0.24 V at 120 h, and energy consumption rose from 84.79 kWh/t to 98.15 kWh/t. These increases were due to the thickening of the anode slime layer over time, which may have led to the formation of a passivation or oxide layer on the electrode surface, impeding metal ion reactions, worsening contact between the anode and electrolyte, increasing resistance, and enhancing electrode polarization, thereby raising cell voltage and energy consumption. Additionally, the accumulation of impurities in the electrolyte over time and their adsorption or involvement in side reactions at the cathode, which consumed some current, contributed to the decline in current efficiency with extended electrolysis.

To elucidate the elemental distribution during the electrolytic refining of crude solder, the cathode deposits were analyzed by ICP-OES (Table S3), and the anode slime composition was determined as shown in Table S6. The changes in electrolyte composition before and after electrolysis (Figure 7c), along with the mass of cathode deposits, anode slime, and electrolyte volume during the process (Table S7), allowed for the determination of elemental distribution behavior among the cathode, anode slime, and electrolyte after refining, as summarized in Table S8 and Figure 7e. From the results, it can be observed that 81.2% of Sn and 75.2% of Pb were deposited on the cathode, while over 93.3% of Sb, Bi, Ag, Cu, and As were enriched in the anode slime. The anode slime was easily removable by scraping, facilitating the subsequent recovery of valuable metals. Additionally, more than 90.6% of In and Al accumulated in the electrolyte. This demonstrates an efficient separation of Sn and Pb from impurities during the electrolytic refining process.

## 4. Conclusions

This study addresses the issues associated with the conventional use of bone glue and β-naphthol in a H_2_SiF_6_ system for solder electrolytic refining, which include complex and unstable electrolyte composition, coarse and non-dense crystallization of cathode solder, low current efficiency, and high energy consumption. We conducted a collaborative optimization study of additives and process parameters in solder electrolytic refining.

(1) The optimal process parameters were determined as follows: an H_2_SiF_6_ concentration of 200 g/L, a current density of 100 A/m^2^, Sn^2+^ and Pb^2+^ concentrations of 50 g/L and 20 g/L, respectively, and a temperature of 35 °C.

(2) Under the optimal conditions, with dynamic replenishment of additives every 24 h, the electrolysis was carried out for 120 h. The current efficiency reached 97.8%, the average cell voltage was 0.24 V, and the energy consumption was 98.15 kWh/t. Compared to the H_2_SiF_6_ system using bone glue and β-naphthol, the current efficiency increased by 1.7%, and the energy consumption decreased by 10.07 kWh/t. The main components of the cathode plate were Sn and Pb, with a purity greater than 99.98%. Additionally, as the electrolysis time extended, the cathode plate remained smooth and dense without dendritic growth.

(3) After the completion of electrolysis (120 h), the concentration of Sn in the electrolyte increased by 4.7 g/L, and that of Pb increased by 2.38 g/L. The changes in the electrolyte composition were minimal, indicating its potential for recycling. A total of 81.2% of Sn and 75.2% of Pb were deposited on the cathode plate. Over 93.3% of Sb, Bi, Ag, Cu, and As were enriched in the anode slime, which facilitated the recovery of valuable metals in subsequent stages. Additionally, over 90.6% of In and Al were enriched in the electrolyte.

## Figures and Tables

**Table 1:** Chemical compositions of the crude solder anode used for electrolytic refining (wt %).

Elements	Sn	Pb	Bi	Sb	In	Cu	Ni
Content	74.358	21.178	2.028	1.814	0.259	0.178	0.0598
**Elements**	**Ag**	**As**	**Co**	**Zn**	**Fe**	**Cd**	**Al**
Content	0.0527	0.0383	0.0027	0.0029	0.002	0.001	0.0007

## Data Availability

The original contributions presented in this study are included in the article. Further inquiries can be directed to the corresponding authors.

## Supplementary Materials

The following are available online at https://www.mdpi.com/article/10.3390/ma18174122/s1, Table S1: Parameter values of φdep, φpc, and φCO of tin and lead deposition at different gelatin concentrations, Table S2: Parameter values of φdep, φpc, and φCO of tin and lead deposition at different sodium lignosulphonate concentrations, Table S3: Element content of the cathode in solder electrolytic refining, Table S4: Comparative analysis on the MSA/H2SiF6, Table S5: The changes in current efficiency, cell voltage, and energy consumption without replenishment of additives, Table S6: element composition of anode slime in enlargement experiment, Table S7: Cathodic deposition quality, anode slime quality, and electrolyte volume during electrolysis, Table S8: The distribution balance table of elements in cathode, anode slime, and electrolyte after electrolytic refining, Figure S1: Cell voltage of crude solder under different conditions: (a) Sn2+/Pb2+ concentration; (b) H2SiF6 concentration; (c) current density; and (d) temperature, Figure S2: The morphology of the cathode surface changes with the extension of electrolysis time for (a) 24 h and (b) 48 h, Figure S3: XPS spectra of (a) C 1s, (b) O 1s, (c) Sn 3d, and (d) Pb 4f of the cathode plate, Figure S4: The composition changes of electrolyte after multiple cycles of use, Figure S5: Variation of cell voltage with electrolysis time.
