# Supplementary material for: Efficient Electrolytic Refining of Crude Solder Assisted by Additives in a Fluosilicic Acid System
Source: Materials (Basel). 2025 Sep 2;18(17):4122. doi: 10.3390/ma18174122 (PMC12429588; doi:10.3390/ma18174122)
Supplement: Supplementary file 1 [file materials-18-04122-s001.zip › materials-3754116-supplementary.pdf]

Efficient Electrolytic Refining of Crude Solder Assisted by Additives in a Fluosilicic Acid System

Yuantao Yang [1,2,#], Zhaoyi Wang [1,2,#], Xuanbing Wang [1,2], Wanli Xu, Haibin Yuan, Qingdong Liu, Ruidong Xu[1,2,*] and Linjing Yang [1,2,*]

Correspondence: rdxupaper@aliyun.com (R.X.); eslinjingyang@kust.edu.cn

Experimental method:

The electrolyte is preheated in the electrolytic cell at a constant temperature and with a circulating liquid flow rate. Once the predetermined temperature is reached, the power supply is connected, and the cell voltage is recorded at regular intervals. The mass of the cathode sample is measured before and after the electrolytic refining process to calculate the cathode current efficiency and energy consumption. Unless otherwise specified, the electrolyte circulation rate is maintained at 50 mL/min, and the electrolytic refining duration is 4 hours. During the whole experiment, the system operates as an open system under environmental pressure.

During the electrolytic refining process, key parameters such as cathode current efficiency, cell voltage, and energy consumption are primarily investigated. Prior to the experiment, the electrolyte is preheated to the set temperature. The circulation of the electrolyte and the power supply are initiated simultaneously. The mass of the cathode plate is measured before and after the electrolytic refining to determine the cathode current efficiency and energy consumption.

Cathodes were made from refined solder plates (2.2×2.2 cm^2 for small-scale experiments and 10.5×11.5 cm^2 for scaled-up experiments), while anodes were fabricated from crude solder (2×2 cm^2 for small-scale experiments and 9.5×10.5 cm^2 for scaled-up experiments).

Supplemental Table

Table S1. Parameter values of φ_{dep}, φ_{pc}, and φ_{CO} of tin and lead deposition at different gelatin concentrations.

GE g/L	φ_{dep} V	φ_{pc} V	j_{pc} mA/cm^2	φ_{CO} V	φ_{pa} V	j_{pa} mA/cm^2
0.1	−0.452	−0.520	41.9	−0.418	−0.332	96.8
0.2	−0.459	−0.565	39.5	−0.418	−0.337	85.1
0.3	−0.461	−0.588	40.5	−0.418	−0.341	77.1
0.4	−0.474	−0.592	39.2	−0.417	−0.343	75.9
0.5	−0.476	−0.609	39.8	−0.417	−0.347	68.7

Table S2. Parameter values of φ_{dep}, φ_{pc}, and φ_{CO} of tin and lead deposition at different sodium lignosulphonate concentrations.

SL<br>g/L	φ_{dep}<br>V	φ_{pc}<br>V	j_{pc}<br>mA/cm^2	φ_{CO}<br>V	φ_{pa}<br>V	j_{pa}<br>mA/cm^2
0.1	−0.528	−0.600	45.6	−0.488	−0.424	61.7
0.2	−0.533	−0.616	43.7	−0.487	−0.429	47.7
0.3	−0.537	−0.628	43.6	−0.487	−0.432	40.3
0.4	−0.539	−0.635	44.2	−0.487	−0.433	36.8
0.5	−0.544	−0.645	44.8	−0.483	−0.434	28.4

Table S3. Element content of the cathode in solder electrolytic refining.

Elements	Sn	Pb	Bi	Sb	In	Cu	Ni
Content	74.90	25.08	0.0012	0.0030	$<$0.0005	$<$0.0005	$<$0.0005
Elements	Ag	As	Co	Zn	Fe	Cd	Al
Content	0.00069	0.0019	$<$0.0005	$<$0.0005	$<$0.0005	$<$0.0005	$<$0.0005

Table S4. Comparative analysis of MSA/H_2SiF_6.

	price	saturation vapor pressure/kPa	boiling point/°C	environmental impact
MSA	~RMB 10,000/t	0.13	167	Readily biodegradable, low toxicity
H_2SiF_6	~RMB 4,000/t	27.5	108.5	Fluoride waste requires neutralization

Table S5. The changes in current efficiency, cell voltage, and energy consumption without replenishment of additives.

Electrolysis time/h	Current efficiency/%	Cell voltage/V	Energy consumption/kWh/t
24	99.01	0.21	84.83
48	98.4	0.23	93.58

Table S6. Element composition of anode slime in enlargement experiment.

Elements	Sn	Pb	Bi	Sb	In	Cu	Ni
Content	33.21	12.77	25.33	23.43	0.30	1.84	0.61
Elements	Ag	As	Co	Zn	Fe	Cd	Al
Content	0.23	0.26	0.023	0.0028	0.035	0.0054	0.0021

Table S7. Cathodic deposition quality, anode slime quality, and electrolyte volume during electrolysis.

cathodic deposition quality/g	anode slime quality/g	electrolyte volume/L
177.2	21.6	5

Table S8. The distribution balance table of elements in cathode, anode slime, and electrolyte after electrolytic refining.

	cathode plate /g	anode slime /g	electrolyte /g	cathode plate /%	anode slime /%	electrolyte /%
Sn	132.7200	7.1700	23.5000	81.2290	4.3883	14.3828
Pb	44.4400	2.7500	11.9000	75.2073	4.6539	20.1388
Bi	0.0021	5.4700	0.0009	0.0384	99.9452	0.0164
Sb	0.0053	5.0600	0.0077	0.1045	99.7447	0.1508
Cu	0.0009	0.3900	--	0.2267	99.7733	--
Ag	0.0012	0.0430	--	2.7589	97.2411	--
As	0.0033	0.0560	0.0007	5.5000	93.3333	1.1667
In	0.0009	0.0560	0.5500	0.1460	9.2274	90.6266
Al	0.0009	0.0005	0.0945	0.9245	0.4696	98.6060

Supplemental Figures

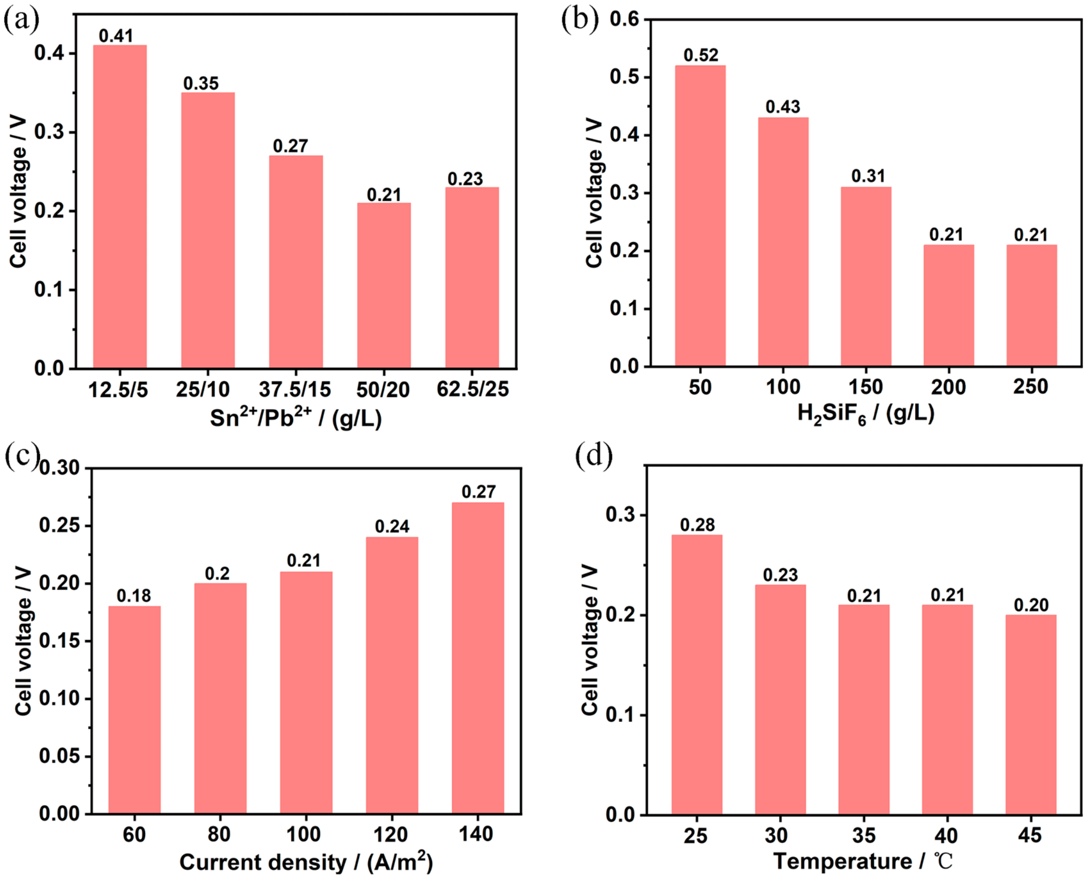

Figure S1. Cell voltage of crude solder under different conditions.

(a) Sn^{2+}/Pb^{2+} concentration; (b) H_2SiF_6 concentration; (c) current density; (d) temperature.

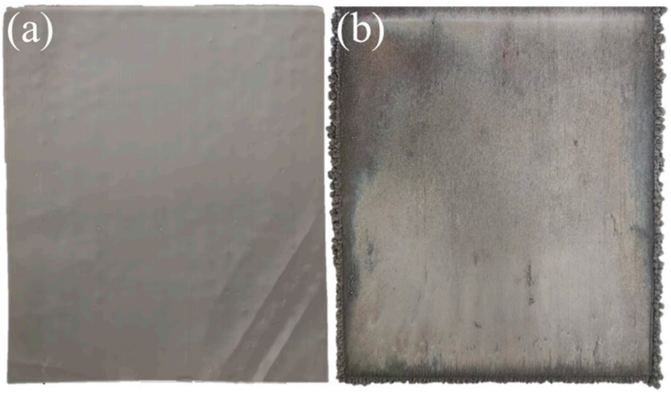

Figure S2. The morphology of the cathode surface changes with the extension of electrolysis time for (a) 24 h and (b) 48 h.

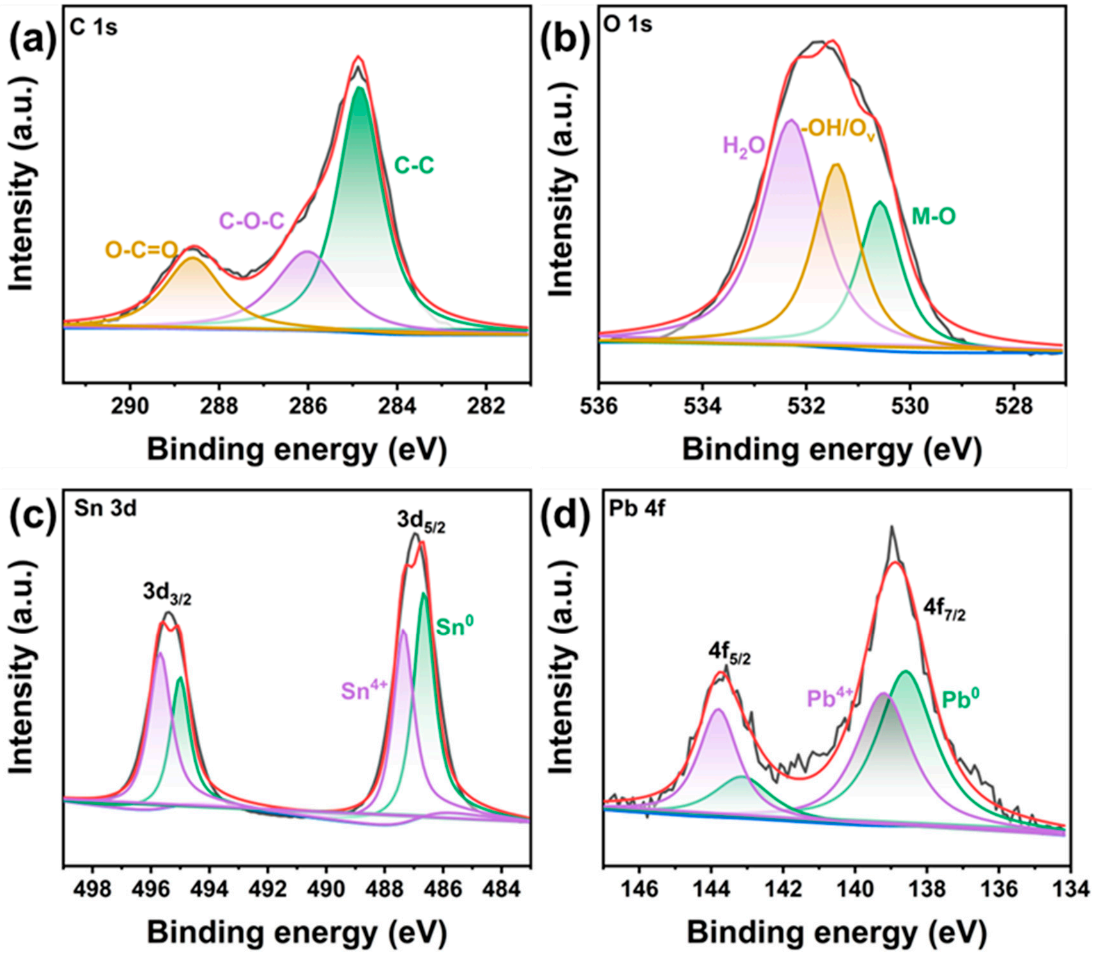

Figure S3. XPS spectra of (a) C 1s, (b) O 1s, (c) Sn 3d, and (d) Pb 4f of the cathode plate.

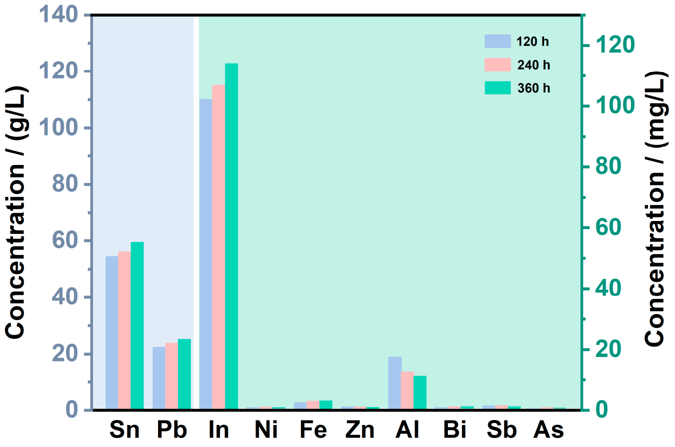

Figure S4. The composition changes of electrolytes after multiple cycles of use.

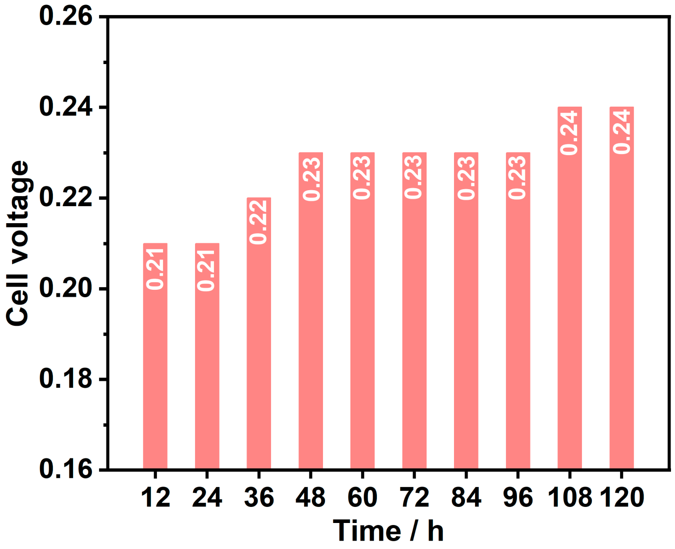

Figure S5. Variation of cell voltage with electrolysis time.
